# *Drosophila melanogaster* as a model arthropod carrier for the amphibian chytrid fungus *Batrachochytrium dendrobatidis*

**DOI:** 10.1371/journal.pone.0307833

**Published:** 2024-07-24

**Authors:** Alyssa M. Byer, Kaylie M. Nguyen, Tatum S. Katz, Renwei Chen, Cheryl J. Briggs

**Affiliations:** Ecology, Evolution, and Marine Biology Department, University of California, Santa Barbara, California, United States of America; Shiv Nadar University, INDIA

## Abstract

The fungal pathogen *Batrachochytrium dendrobatidis* (*Bd*) causes the disease amphibian chytridiomycosis, which has contributed to population declines in many species of amphibians throughout the world. Previous observational studies have shown that nematodes, waterfowl, lizards, other dipterans, and crayfish have properties which may allow them to harbor and spread *Bd*; therefore, we sought to determine the carrier capabilities of invertebrates to a further extent in a laboratory setting. We use the insect *Drosophila melanogaster* as a model organism to quantify the potential relationship between insects and *Bd*. Our findings show that *D*. *melanogaster* can test positive for *Bd* for up to five days post-exposure and can transmit *Bd* to conspecifics without suffering mortality. Insects of various types interact with the amphibian habitat and amphibians themselves, making this a potentially important route of transmission between amphibians and of dispersal across the environment.

## Introduction

The infectious disease chytridiomycosis, caused by the fungal pathogen *Batrachochytrium dendrobatidis* (*Bd*), has contributed to global amphibian population declines and species extirpations [1–3]. *Bd* infects the keratinized skin of an amphibian host, disrupting the flow of electrolytes across the cell membrane, ultimately leading to cardiac arrest [4]. While much of the research on *Bd* has focused on its effects on amphibian populations, increasing evidence suggests that *Bd* may be a generalist fungal pathogen with non-amphibian hosts, carriers, and reservoirs. Following its discovery, researchers have hypothesized that *Bd* may be a sapronotic pathogen, one which may live and reproduce on other organisms and substrates in the environment [5, 6], and theoretical work supports this hypothesis [7]. Alternative hosts, carriers, and reservoirs can help maintain a pathogen in the environment, increasing prevalence and allowing it to drive a focal host species extinct [8]. Traditional disease theory predicts that a pathogen with density-dependent transmission would be eliminated following disease-induced decline of its focal host because there are too few individuals to maintain it in the population—this is known as epidemic fade-out [8, 9]. However, *Bd* has not experienced epidemic fade-out. If *Bd* has non-amphibian hosts, it would explain how *Bd* continues to spread despite extirpation of its amphibian hosts. Furthermore, *Bd* is a member of the phylum Chytridiomycota [6]. Most other chytrids are free-living detritivores or invertebrate parasites, with *Bd* and its sister species (*Batrachochytrium salamandrivorans*) being unique in their ability to infect vertebrates [10].

To evaluate evidence of a given organism’s potential to function as a carrier, and thus to understand its contribution to the disease dynamics of a focal host population, we must first define what can be considered a carrier. Due to the high variation in the use of the term vector [11], we have instead chosen to use the term carrier. We assert that a carrier of *Bd* is an organism that can move *Bd* from one location to another (a location being either an amphibian, alternative host, or environmental reservoir such as pond water) and can do so without suffering extreme deleterious effects. Therefore, our definition will include organisms which function only as “mechanical vectors” [11], meaning they do not necessarily become infected themselves. We include that the carrier must not suffer extreme negative effects of *Bd* because that could hinder its ability to move the pathogen through the environment. Organisms which suffer illness or mortality as a result of exposure to *Bd* would be considered “alternative hosts” under our definition, and this is the main difference between the terms. Accordingly, to be a carrier under our definition, the organism must meet two major criteria: it must act as a vehicle for moving *Bd* between two surfaces, and it must not suffer from exposure to *Bd*.

A wide variety of studies have been conducted to elucidate *Bd*’s functions on non-amphibian hosts. The majority of studies utilize DNA detection to screen for *Bd* presence [12–20], while a subset solely or additionally leverage histology or laboratory analyses to understand *Bd*’s interaction with their chosen potential alternative host [12, 15, 19–25]. The first finding to provide evidence that *Bd* may have non-amphibian hosts, environmental reservoirs, or carriers examined if *Bd* could persist on sterile pond substrate or bird feathers [22]. Fascinatingly, they found that *Bd* could persist for up to 3 months in sterile pond substrate and survived three hours of drying time on sterile bird feathers [22]. The authors were successfully able to culture live *Bd* from these substrates after the experiment, providing a level of evidence above detection of *Bd* DNA that these substrates could function as environmental reservoirs or, in the case of waterfowl, carriers, for *Bd*. Following this study, a body of literature was developed which sought to detect *Bd* DNA on a variety of organisms: wild snakes and lizards [13, 14], wild waterfowl [15] and waterfowl museum specimens [16]; wild and farmed crayfish [17], wild midges [18], and mosquitoes [12]. Garmyn and colleagues [15] expanded on their DNA detection by performing a series of laboratory experiments wherein they demonstrated that *Bd* chemotaxes to keratinized toe-clippings of waterfowl. One notable study not only detected *Bd* DNA on wild crayfish, but also showed positive histological analysis of *Bd* zoospores in the gut of the crayfish and pathogenicity of *Bd* towards crayfish in the laboratory [19]. Oficialdegui and colleagues [20] also showed *Bd* positive histological analysis and DNA detection in crayfish guts, and the specific mechanisms of *Bd* pathogenicity towards crayfish were further explored in [21], providing further evidence that crayfish are an alternative host of *Bd*. Laboratory investigations have shown that *Bd* can infect zebrafish [23] and nematodes ([24]; with controversy, see [26]), and can be transmitted by mosquitoes to sterile agar plates in a laboratory setting [12, 25]. However, *Bd* DNA could not be detected on wild freshwater snails, and the snails and green algae could not be experimentally infected with *Bd* in a laboratory setting [27].

While evidence builds that *Bd* may have non-amphibian hosts and environmental reservoirs, few studies have been done to quantify and parameterize these interactions (but see the work done on nematodes, i.e. [24, 26]), a critical step for producing more accurate models of the *Bd*-amphibian system. Quantifying and understanding the importance of these non-amphibian hosts and vectors is key to our ability to model, control, and manage *Bd*-related amphibian population declines. Therefore, we sought to develop a series of experiments which could be applied broadly to potential invertebrate *Bd* hosts using the classic model organism *Drosophila melanogaster*. This species has been used as a model organism since the early 20^th^ century [28] and is very commonly used as a feeder animal for captive amphibian colonies. We selected it due to these qualities and to act as a model organism for other short-lived insects which may interact with multiple, otherwise isolated, amphibian habitats. Here, we present a series of experiments that demonstrate that *D*. *melanogaster* can be inoculated with and maintain *Bd* as well as transmit *Bd* to conspecifics, without suffering any mortality by the pathogen. We also estimate the function for *D*. *melanogaster* to *D*. *melanogaster* transmission in order to quantify the interactions between this potential model arthropod carrier and *Bd*.

## Materials and methods

Culture of *Bd*. *Bd* was cultured on 1% tryptone agar plates one week prior to each experiment. Inoculum consisted of a cocktail of four pathogenic *Bd* strains isolated from *Rana sierra* or *R*. *muscosa* in the California Sierra Nevada mountain range: TST77, CJB4, CJB5-(2), and CJB7 [29, 30]. Concentrations of *Bd* were counted using a hemocytometer and *Bd* zoospore viability was visually confirmed via microscopy. Viable zoospores are motile, spherical, and appear three-dimensional under the microscope while nonviable zoospores do not move and are generally dark, small, and irregularly shaped.

Fly stocks. Nonsterile *Drosophila melanogaster* (obtained from Josh’s Frogs, https://joshsfrogs.com/) were maintained in plastic containers at room temperature, containing pre-mixed fruit fly media (Josh’s Frogs) and wood wool (“excelsior”, Josh’s Frogs). Flies were transferred to new containers approximately every two weeks. Fruit fly preliminary experiments showed that pre-mixed fruit fly media, which contains antifungal substances, prevented *Bd* growth and inhibited infection of *D*. *melanogaster* (Supporting information). Therefore, we used a homemade media consisting of potato starch, sugar, and yeast that utilized dilute vinegar as an antimicrobial and did not inhibit *Bd* growth for experiments (Supporting information). Flies were moved to vials with homemade fruit fly media 24 hours prior to all experiments.

Inoculation of *D*. *melanogaster* with *Bd*. To determine if *D*. *melanogaster* can possibly act as a carrier for *Bd*, we first sought to examine if *D*. *melanogaster* can uptake *Bd* and if we could later detect *Bd* DNA on the flies. Flies were separated by sex and were exposed to either 10^7^ *Bd* zoospores, 10^7^ heat-killed *Bd* zoospores, or sterile deionized water in groups of 10 each on 1% tryptone agar plates (S2 Table). The treatment solutions (live *Bd*, heat-killed *Bd*, or sterile water) were applied to the plates and allowed to dry down slightly before the flies were added. Plates were sealed with parafilm and incubated for 24 hours at room temperature. During this time, the flies were able to move freely on the plates. The heat-killed *Bd* control was included to determine whether the levels of *Bd* DNA detectable on the flies were comparable for flies exposed to dead and living zoospores. The flies were separated based on sex because it has been shown that flies have X chromosome-linked variation in immune response, which may result in differing fungal load [31].

After 24 hours of incubation at room temperature, flies were transferred in groups of ten from each agar plate to vials containing homemade fruit fly media (Supporting information), where they were observed for up to five days. One vial of flies for each sex and *Bd* treatment was euthanized by freezing at -20°C each day for five days. Five flies from each vial (one experimental unit) were washed with 500μL of PBS buffer per fly for approximately one minute to remove external, passively-adhered *Bd* or *Bd* DNA to determine if the DNA was superficial on the fly or if the detected DNA may represent a true infection. The remaining five flies went directly to a storage vial with no washing (S2 Table).

This experiment was repeated three times: two trials were conducted using all three *Bd* treatments, while one preliminary trial contained only the treatments of live *Bd* and sterile water (n = 2 or 3 experimental units, S2 Table). Flies were stored at -20°C until DNA extraction and qPCR analysis to determine *Bd* presence and load. Five flies (half of each vial) were included in a single DNA extraction, creating a single experimental unit for analysis. To test if *Bd* treatment, washing treatment, day, or sex predicted *Bd* load, a linear mixed-effects model was developed with fly vial as a random effect using the R [32] package lme4, followed by planned comparison t-tests to determine significant contrasts within treatments [33]. This analysis and all statistical analyses were performed at an alpha of 0.05. The protocol for inoculation D. melanogaster with *Bd* can be found at [34].

Optimization of the *Bd* concentration for inoculation. To determine the minimum dose of *Bd* required for inoculation, we inoculated flies following the methods above but with a varying number of zoospores per plate: 0, 10^4^, 10^5^, 10^6^, and 10^7^. Following inoculation, flies were transferred to fly vials without any fruit fly media for an additional 24 hours. Flies were then euthanized at -20° C. Half of the flies were washed with PBS as above prior to DNA extraction to remove external *Bd* to try to discern if Bd is present on the exoskeleton or internally, while the other half was unwashed and went directly into a storage vial (n = 2 experimental units (10 flies total) per sex, washing treatment, and dosage treatment combined, S3 Table). *Bd* load was evaluated with quantitative PCR, with five flies (one experimental unit) per reaction. To test if dosage, washing, or sex treatments produced significantly different mean *Bd* loads, a three-way ANOVA was run followed by a Tukey-Kramer test to identify the significantly different groups [32].

Mortality of *D*. *melanogaster* caused by *Bd*. To determine if *Bd* has negative effects on *D*. *melanogaster* survival, a mortality assay was conducted. Flies were separated by sex and incubated for 24 hours on 1% tryptone agar plates in groups of five with the following treatments: 10^6^ *Bd* zoospores per fly, *Bd* supernatant from 10^6^ zoospores per fly, and sterile deionized water (S4 Table). *Bd* supernatant was included because evidence suggests that *Bd* supernatant interferes with cell junctions and immune cell function in amphibians [35], therefore we sought to determine if the supernatant similarly had deleterious effects on flies. The supernatant was prepared by creating suspensions of 200 x 10^6^ *Bd* zoospores in sterile deionized water, spinning them down in a centrifuge at 2000rpm for 10 minutes, and decanting the supernatant. Therefore, the supernatant contained any *Bd* zoospore products but no zoospore cells. A volume of 40mL of *Bd* supernatant was recovered from 200x10^6^ *Bd* zoospores, and each plate was dosed with 0.5mL of the supernatant product.

Following incubation, flies were transferred in groups of five from each plate to fly vials containing homemade fruit fly media using microspatulas and forceps, sterilizing the instruments between each individual by soaking them in 70% ethanol and then flaming (Supporting information). Fly deaths were recorded daily until the end of the experiment (day five). To determine if treatment groups had significantly different mortality, Kaplan-Meier survival curves were fit to the data and a Log-Rank test comparing the curves was run using the R package survival [36].

Transmission of *Bd* among *D*. *melanogaster*. To determine if *D*. *melanogaster* could transmit *Bd* to conspecifics, a transmission experiment was conducted. Flies were split into two groups, susceptible and inoculated. Flies in the inoculated group were inoculated in groups of 10 per agar plate containing 10^7^ zoospores (which was demonstrated to produce 100% *Bd*+ qPCR results in the dosage optimization experiment), following the methods described above. Flies in the susceptible group were not inoculated.

After inoculation, inoculated and susceptible flies were combined in vials ranging in fly density and initial infection prevalence (S2 Fig, [37]). Fly densities ranged from as low as two flies per vial to 34 flies, and initial inoculation prevalence was either 12.5%, 25%, or 50%. Replicates were not included, rather, we leveraged the experimental design of Tompros and colleagues [37] whose design maximizes the range of density and prevalence combinations, rather than maximizing repetition at a narrower range, for model fitting. Susceptible and inoculated flies were non-differentiable. To account for this, we assumed that all inoculated flies were *Bd*+ and tested flies for *Bd* DNA individually. Therefore, if a treatment group yielded a higher number of inoculated flies than we initially introduced, we assumed the additional *Bd*+ flies had *Bd* transmitted to them.

Flies remained in vials, moving around freely at room temperature for 24 hours. Flies were subsequently euthanized by freezing at -20°C and were moved into individual reaction tubes (one fly per reaction) for DNA extraction and qPCR for *Bd*. We followed the approach of Rachowicz and Briggs [9] to determine the best-fit transmission function using a maximum likelihood approach and AICc in MATLAB [38, 39].

DNA extraction from *D*. *melanogaster* and qPCR for *Bd*. DNA extraction was performed on either pooled groups of five flies (inoculation experiment, dosage optimization experiment), or on a single fly (transmission experiment). *D*. *melanogaster* were homogenized with 100μl of 1 mm silica beads and 180μl ATL buffer (DNeasy Blood & Tissue Kit, QIAGEN) for 3 minutes at 2400 rpm using a mini beadbeater. Following homogenization, samples were incubated with proteinase K (diluted 1:11 in ATL buffer) at 37°C for 8 hours. DNA was subsequently purified using the DNeasy Blood & Tissue Kit (QIAGEN) following manufacturer’s instruction. Quantification of *Bd* DNA on *D*. *melanogaster* was analyzed using real-time PCR (ABI7300 Sequence Detection System, Applied Biosystems). A total 25 μL of PCR reaction consists of 5 μL of DNA, 1X SensiFAST Probe Hi-ROX Kit (Meridian Bioscience), 1 X TaqMan^™^ Exogenous Internal Positive Control Reagents (Appliedbiosystems), probe, forward and reverse primers as described in [40]. Due to monetary and temporal limitations and based on the work of [41], PCR reactions were performed in singlicate. Laboratory cultured *Bd* zoospores served as standard control and results are reported in zoospore equivalents per fly (ZE). The protocol for DNA extraction of *Bd*-inoculated D. melanogaster can be found at [42].

## Results

### Inoculation of *D*. *melanogaster* with *Bd*

**Fig 1:**
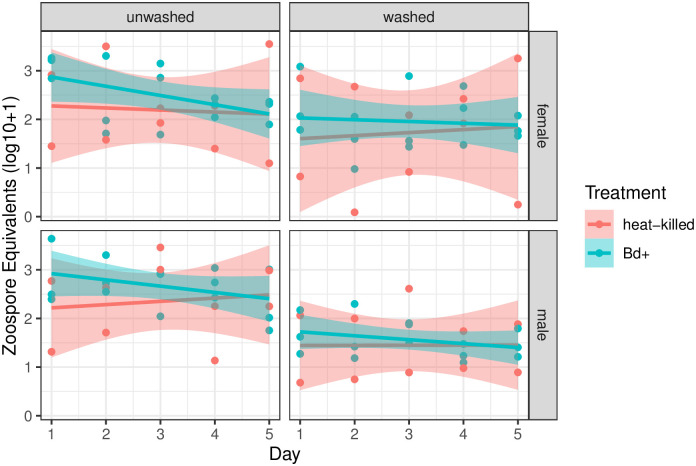
*Drosophila melanogaster* were inoculated with *Bd* or heat-killed *Bd* and sacrificed each day for five days. Fit lines represent a linear model and shaded ribbons represent 95% confidence intervals. Each datapoint represents one experimental unit, the pooled DNA of five flies, reported in zoospore equivalents per fly.

All experimental units (five flies per DNA reaction) in both the heat-killed and *Bd*-positive treatments were positive for *Bd*, with no significant difference in load between the treatments (Welch’s t(65.704) = -1.41, p = 0.16, Fig 1), indicating that DNA from dead *Bd* spore is as detectable as DNA from live spore. No experimental units in the negative control treatment were positive for *Bd*. Furthermore, days post exposure and sex had no effect on *Bd* load (p > 0.05). These results indicate that *Bd* may be maintained on flies but does not appear to reproduce on them within five days. Furthermore, there may not be sex differences between flies in relation to *Bd* exposure. Washed flies, however, had significantly lower loads (Welch’s t(79) = 6.54, p = -5.9 x 10^9^, S2 Fig), indicating that a significant amount of *Bd* was passively adhered externally to the fly.

### Optimization of the Bd concentration for inoculation

**Fig 2:**
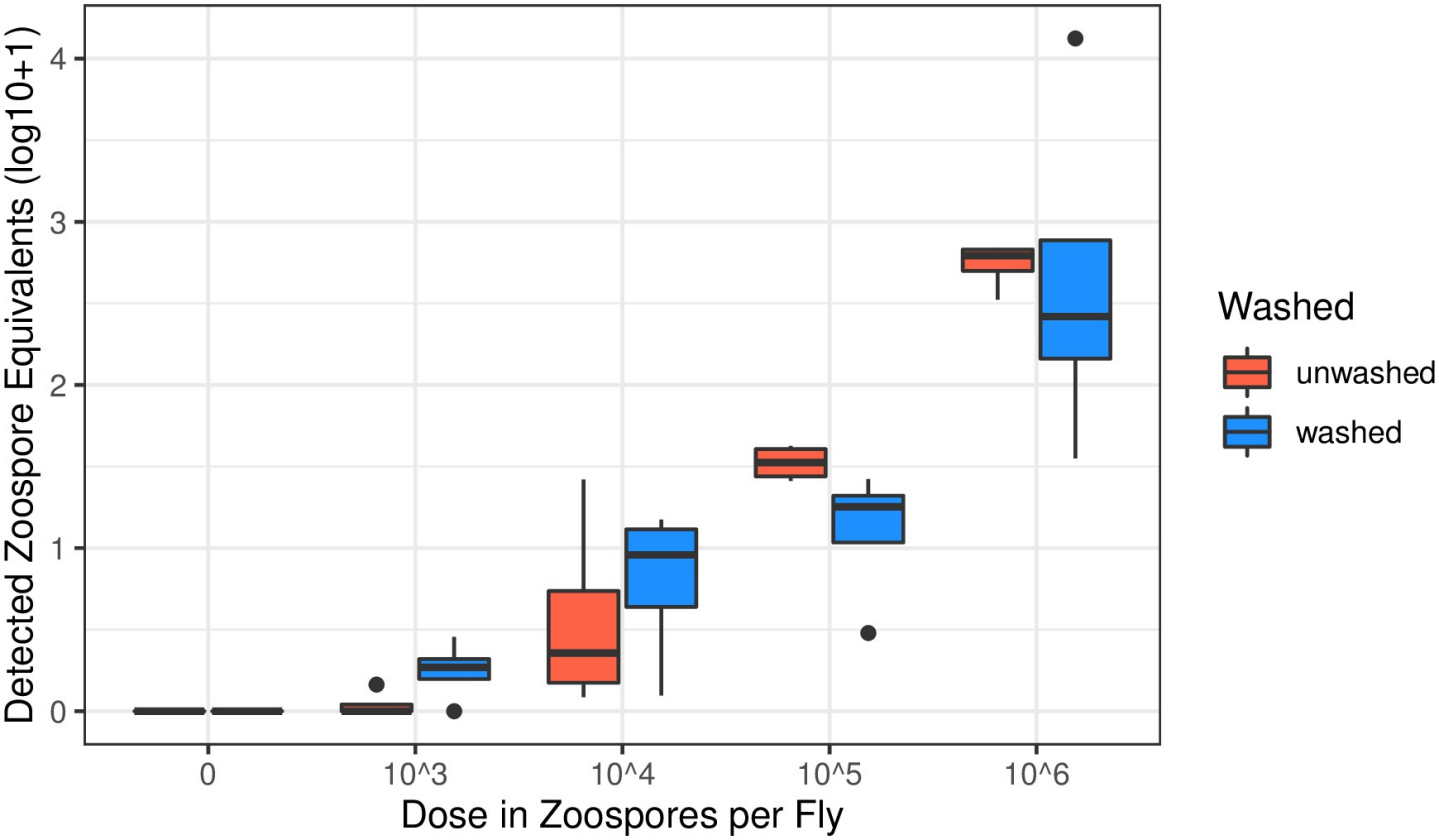
*Drosophila melanogaster* were inoculated with *Bd* at a range of concentrations. Each datapoint represents one experimental unit, the pooled DNA of five flies, reported in zoospore equivalents per fly. There was no significant difference between sexes therefore results are pooled across sex.

All experimental units (five flies per unit) in all treatment groups (10^3^, 10^4^, 10^5^, or 10^6^ zoospores per fly), except those dosed with 10^3^ zoospore per fly, were *Bd* positive; within the 10^3^ zoospore dose group, four of eight experimental units (pooled by sex and washing treatment) were *Bd* positive, indicating that doses as low as 10^3^ zoospores per fly can produce *Bd* positive qPCR results. No flies from the negative control group tested positive for *Bd*. The interaction of dosage, washing treatment, and sex, or any combination thereof, was not significant (p>>0.05), thus results are reported on the ANOVA with no interactions. Inoculation dose was a significant predictor of *Bd* load (three way ANOVA, f(4, 13) = 48.53, p = -2.27 x 10^13^), while sex and washing treatment were not significant predictors (p > 0.05, Fig 2). These findings further support a lack of sex differences in relation to *Bd* exposure, but contrast the previous experiment in washing findings. This may indicate that in this experiment, *Bd* DNA or spores were more strongly adhered to the fly, as washing did not decrease loads.

### Mortality of *D*. *melanogaster* caused by *Bd*

**Fig 3:**
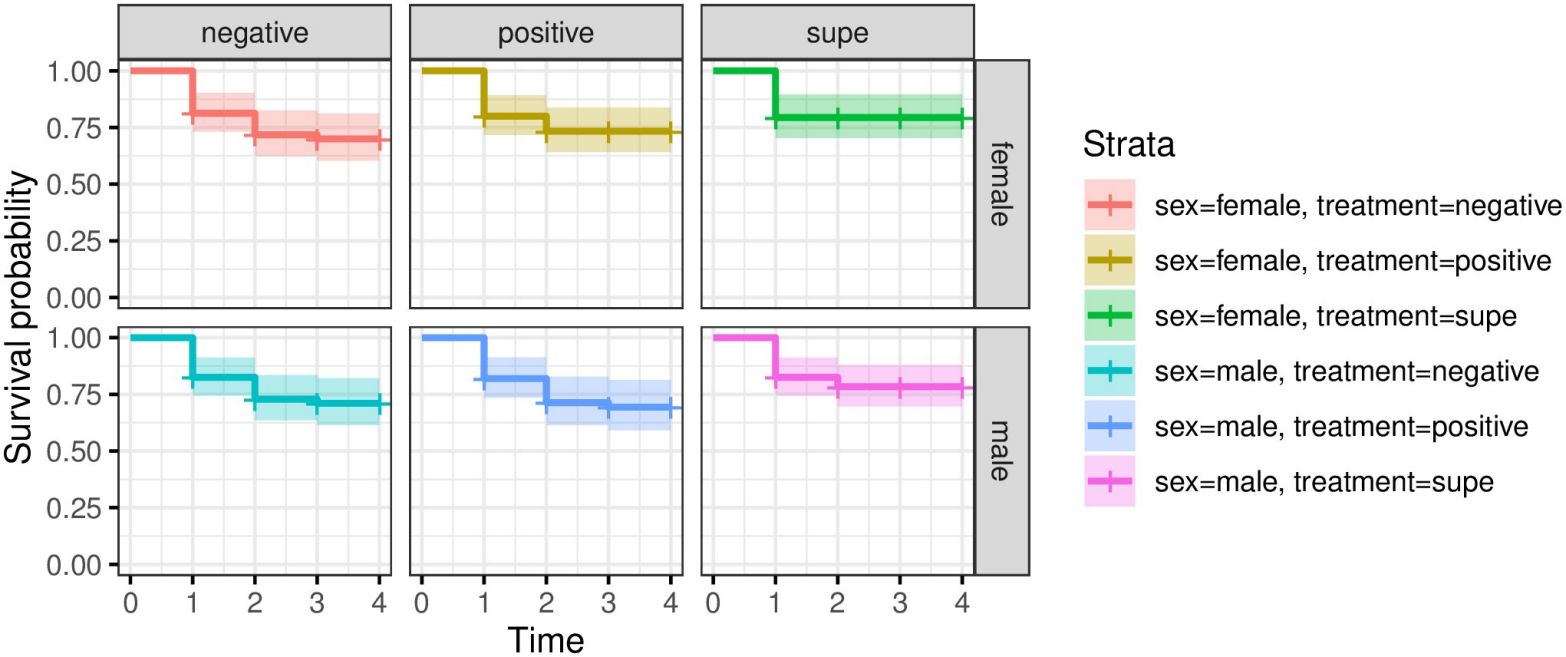
*Drosophila melanogaster* were split by sex inoculated with either live *Bd* spore, *Bd* supernatant, or water (as a negative control) and monitored for five days for mortality. Plot shows Kaplan-Meier survival curves for each of the experimental groups.

We found no significant difference in survival curves across any of the treatment groups (*χ*^2^(23) = 18.6, p-value = 0.70), indicating there was no effect of sex, fly vial, or *Bd* treatment in determining fly mortality within the timeline of the experiment (Fig 3). This demonstrates that *D*. *melanogaster* may not suffer mortality due to *Bd* within five days.

### Transmission of Bd among *D*. *melanogaster*

During the process of transferring inoculated and susceptible flies to their experimental treatment groups, the 20 fly density groups for both 12.5% and 50% inoculation prevalence were lost. Additionally, rather than an n = 32, 25% prevalence group, an n = 34, 24% prevalence group was created (S2 Fig). Data were fit to various transmission functions: constant risk, density dependent, frequency dependent, power, asymptotic, and negative binomial as discussed in [9, 43]. Estimates for function parameters and AICc values are given in Table 1. The function with the lowest AICc value was the power function (AICc = 62.51), and ⊗AICc to the next-best function (density dependence) was 1.19, indicating little to no discrimination between these two transmission types. However, there was moderate discrimination between the best function (power) and all other functions (⊗AICc > 2). These results indicate that *D*. *melanogaster* may transmit *Bd* between conspecifics in a density-dependent manner.

**Table 1:** Transmission function results.

Transmission Type	Function^a^	Parameter Estimates	AICc	⊗AICc
**Power**	*βSI* ^*q*^	*β* = 0.0012	62.51	0.00
		*q* = 2.16		
**Density Dependent**	*βSI*	*β* = 0.015	63.70	1.19
**Asymptotic**	$\frac{\beta SI}{\left(c+S+I\right)}$	*β* = 0.63	64.90	3.39
		*c* = 18.51		
**Negative Binomial**	$k\;ln\left(1+\frac{\beta I}{k}\right)S$	*β* = 8.79 × 10^−5^	66.09	3.58
		*k* = 170.99		
**Constant Risk**	*βS*	*β* = 0.097	75.93	13.42
**Frequency Dependent**	*βSI/N*	*β* = 1.00	97.29	34.78

^a^ Where S is the number of susceptible (uninoculated) flies; I is the number of inoculated flies; N is the total number of flies such that S + I = N; *β* is the transmission parameter with units time^-1^ (where one timestep is one day); and q, c, and k are dimensionless constants. Notation ln represents the natural log of the following equation within parentheses.

## Discussion

Understanding the full ecology of the *Bd*-amphibian disease system is critical to our ability to manage *Bd*-related amphibian die offs. Invertebrates have long been suspected to be *Bd* hosts or carriers, yet few laboratory-based studies have been done to quantify the parameters of the relationship. Under our definition, carriers must be able to move *Bd* through the environment and not suffer extreme negative effects as a result, while a host is any organism that is infected by *Bd*. Based on previous work and our definition of a carrier in the *Bd* system, we sought to develop a series of experiments using an arthropod model organism, *Drosophila melanogaster*, to estimate important parameters necessary for modeling disease dynamics. Taken together, the results of our experiments suggest that not only can *D*. *melanogaster* be inoculated with *Bd* at ecologically-relevant loads and not suffer immediate mortality, but can even transmit *Bd* to conspecifics. No differences were found between sexes, suggesting *D*. *melanogaster* sexes do not differ in their response (if any) to *Bd*. In the inoculation and dosage-dependency experiments, flies were *Bd* positive even after thorough washing, indicating that the positive qPCR result is likely not due to passively adhered *Bd* DNA, but instead viable *Bd* zoospores that may be adhered internally or externally to the fly.

Washing the flies in PBS solution only significantly reduced *Bd* loads in the inoculation experiment, rather than removing *Bd* completely, indicating the *Bd* DNA detected is more than passively attached to the flies. However, as flies in the inoculation experiment dosed with heat-killed *Bd* and subsequently washed still returned positive *Bd* qPCR results, this indicates that even dead *Bd* has the ability to stay attached to the organism through vigorous washing. This provides evidence that detecting *Bd* DNA is not directly indicative of viable *Bd*, much less *Bd* infection. Many other studies have successfully detected *Bd* DNA on various substrates in the amphibian habitat [12, 13, 15, 19, 24, 44], yet these may not indicate true carriers or reservoirs of *Bd* based on our findings. Rather, they could be the result of shed, dead *Bd* spore from an infected amphibian. This result is further supported by the lack of a significant difference between qPCR results of live vs. heat-killed *Bd* in the inoculation experiment. Further research should seek to develop methods to determine if detected *Bd* DNA represents viable, infectious zoospores or simply dead *Bd* adhered to an organism or substrate.

In the dosage-dependency experiment, we found that flies could be inoculated with *Bd* at doses as low as 1000 zoospores per fly and we obtained 100% *Bd* positive results at 10,000 zoospores per fly and greater. In natural settings during an outbreak, a single frog may produce a swab with tens of thousands of zoospores [45, 46] and a milliliter of water can contain approximately 1000 zoospores [47]. Flies in natural settings come into contact with water and amphibians to gain moisture, representing a possible transmission pathway between not only bodies of water in a system, but between amphibians themselves. Exposure to both *Bd* and *Bd* supernatant had no effect on fruit fly mortality within the five-day period of the experiment, indicating that if the flies are experiencing negative effects due to *Bd* exposure, they are able to tolerate it well for a long enough time to move *Bd* across the environment. Whether the flies are truly infected by *Bd* or simply acting as a vehicle of transmission is beyond the scope of this work, yet our findings demonstrate that fruit flies have the potential to be a carrier for *Bd* in natural settings.

While our transmission experiment could not discriminate between a power function or a density-dependent function for transmission, we nonetheless observed transmission between *D*. *melanogaster* in the laboratory setting. This aligns with findings from other amphibian disease studies; Rachowicz and Briggs [9] found that *Bd* transmission in the wild was best described by a density-dependent function. While few studies exist which attempt to quantify transmission functions for amphibian diseases, the inability to confidently discriminate between transmission functions outlines the difficulty in estimation of transmission functions in this system. Despite lack of clarity on transmission function, we have shown that *D*. *melanogaster* can transmit *Bd* to conspecifics which contributes further evidence for the ability of winged insects to act as carriers in the natural environment.

Our study has two major limitations to our conclusions: lack of real-time PCR replicates, and that our study relies s solely on DNA-based *Bd* detection. While replicates were performed at the organismal level, without running our reactions in triplicate we cannot disentangle sources of error from the molecular analyses versus variation in the inoculation process itself. Due to logistical constraints, we referred to the work of [41] who determined that there is no significant loss of accuracy when running qPCR in singlicate versus triplicate. Future work should continue to explore the relevance of arthropods in Bd transmission and explore whether infectious, viable *Bd* can persist on relevant arthropods at ecologically relevant dosages and timeframes. Finally, experiments to determine if wild fruit flies can transmit *Bd* to amphibians would be important to further elucidate the role of insects in pathogen transmission in amphibian populations.

In conclusion, our work deepens the current understanding of arthropods as possible vectors of Bd by examining important parameters of this relationship in an experimental, laboratory setting using D. melanogaster as a model arthropod. Our findings demonstrate that D. melanogaster has the ability to uptake and transmit Bd spores at ecologically relevant dosages and timeframes. This series of experiments can be used to inform mathematical models of Bd transmission dynamics involving insect carriers, or replicated on other potential Bd carriers to enhance our understanding of this important fungal pathogen.

## Supporting information

S1 Appendix Fruit fly media inhibits *Bd* growth. (DOCX)

S1 Fig *Bd* growth is inhibited by pre-mixed fruit fly media. Five variations of homemade fruit fly media containing different concentrations of vinegar and brewer’s yeast (LV, LY, MV, MY, New) were compared to Josh’s Frog’s fruit fly media (Joshs) for inhibition of Bd growth. Josh’s Frog’s fruit fly media produced significantly fewer Bd zoospores than all other treatments. Groups denoted by a different letter indicate significant differences between treatments (p < 0.05).
(PNG)

S2 Fig Transmission experiment design and experimental flow. Experimental design and table were adapted from Tompros et al. 2021. The table on the left shows the total density of flies and the total number of inoculated flies in each vial. Asterisks denote treatments that were lost. In the experimental flow on the right, inoculated flies are represented in red and susceptible flies in yellow. Color indicates inoculation status only, and flies were not marked during the experiment.
(PNG)

S1 Table Fruit fly media recipe treatments. (DOCX)

S2 Table Inoculation experiment. Table rows and columns give treatments, while cell values indicate the number of experimental units (one vial or five flies, their DNA pooled) in that treatment.
(DOCX)

S3 Table Dosage dependency experiment. Table rows and columns give treatments, while cell values indicate the number of experimental units (one vial or five flies, their DNA pooled) used in that treatment.
(DOCX)

S4 Table Mortality assay experiment. Table rows and columns give treatments, while cell values indicate the number of flies used in that treatment. Flies were inoculated in groups of five, and held in fly vials for observation in groups of five.
(DOCX)
