# Supplementary material for: Drosophila melanogaster as a model arthropod carrier for the amphibian chytrid fungus Batrachochytrium dendrobatidis
Source: PLoS One. 2024 Jul 24;19(7):e0307833. doi: 10.1371/journal.pone.0307833 (PMC11268706; doi:10.1371/journal.pone.0307833)
Supplement: S1 Appendix — (DOCX) [file pone.0307833.s001.docx]

**Supporting Information Appendix 1 for *Drosophila melanogaster* is a possible vector of *Batrachochytrium dendrobatidis***

**Fruit fly media inhibits Bd growth.**

**Methods.**

To determine if pre-mixed fruit fly media (Josh’s Frogs Fruit Fly Media) inhibits Bd growth, and to determine an appropriate concentration of vinegar in homemade fruit fly media that would inhibit mold but not Bd growth, we performed a growth study with Bd. For each fruit fly media recipe treatment (Supporting Table 1), a petri dish was prepared with a thin layer of the fruit fly media spread across it. 1ml of Bd stock was pipetted over the media. Plates were allowed to dry, uncovered, for 2 hours and then sealed with parafilm and stored at room temperature for one week. After one week, plates were flooded with sterile deionized water and incubated at room temperature for 30 minutes so sporangia could release zoospores. Zoospores were harvested by pipetting water off the plates. Zoospores were then washed by spinning down in a centrifuge at 2000rpm for 10 minutes, decanting the supernatant, adding sterile deionized water to the tube, resuspending the pellet, and repeating. Zoospore concentrations were determined by hemocytometer and compared between treatments. For statistical analysis, each 1mm x 1mm corner square was used as an observation (n = 4 per treatment). A one-way ANOVA was performed followed by a Tukey-Kramer test to identify significantly different groups.

**Results.**

Bd growth differed significantly between fruit fly media treatments (one-way ANOVA with α = 0.05, F(5, 24) = 350.1, p < 0.001), with the store-bought media producing the significantly lowest Bd growth (Tukey-Kramer with 95% family-wise confidence level, all p-values << 0.001, Supporting Figure 1). No treatments had mold growth after one week.
