# Supplementary figures and images for: Drosophila melanogaster as a model arthropod carrier for the amphibian chytrid fungus Batrachochytrium dendrobatidis
Source: PLoS One. 2024 Jul 24;19(7):e0307833. doi: 10.1371/journal.pone.0307833 (PMC11268706; doi:10.1371/journal.pone.0307833)

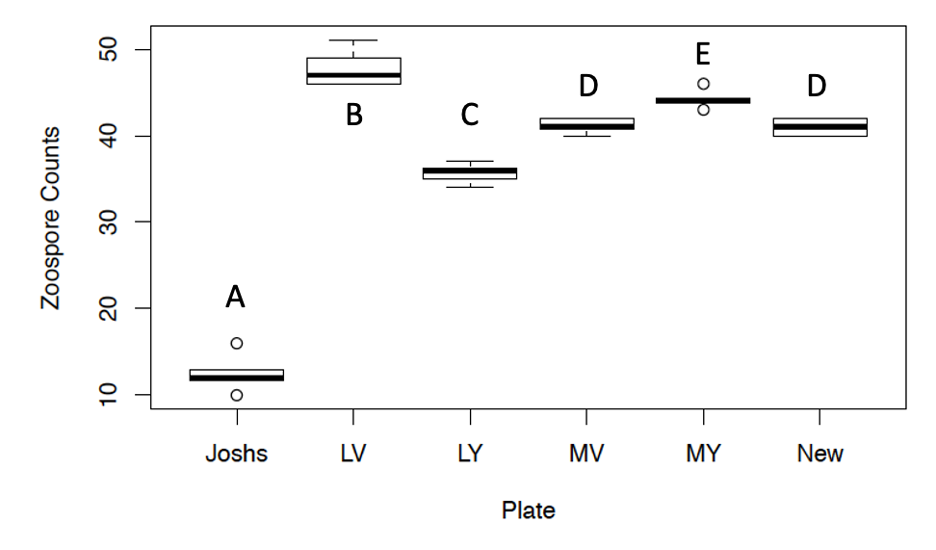

Supplement: S1 Fig — Five variations of homemade fruit fly media containing different concentrations of vinegar and brewer’s yeast (LV, LY, MV, MY, New) were compared to Josh’s Frog’s fruit fly media (Joshs) for inhibition of Bd growth. Josh’s Frog’s fruit fly media produced significantly fewer Bd zoospores than all other treatments. Groups denoted by a different letter indicate significant differences between treatments (p < 0.05). (PNG) [file pone.0307833.s002.png]

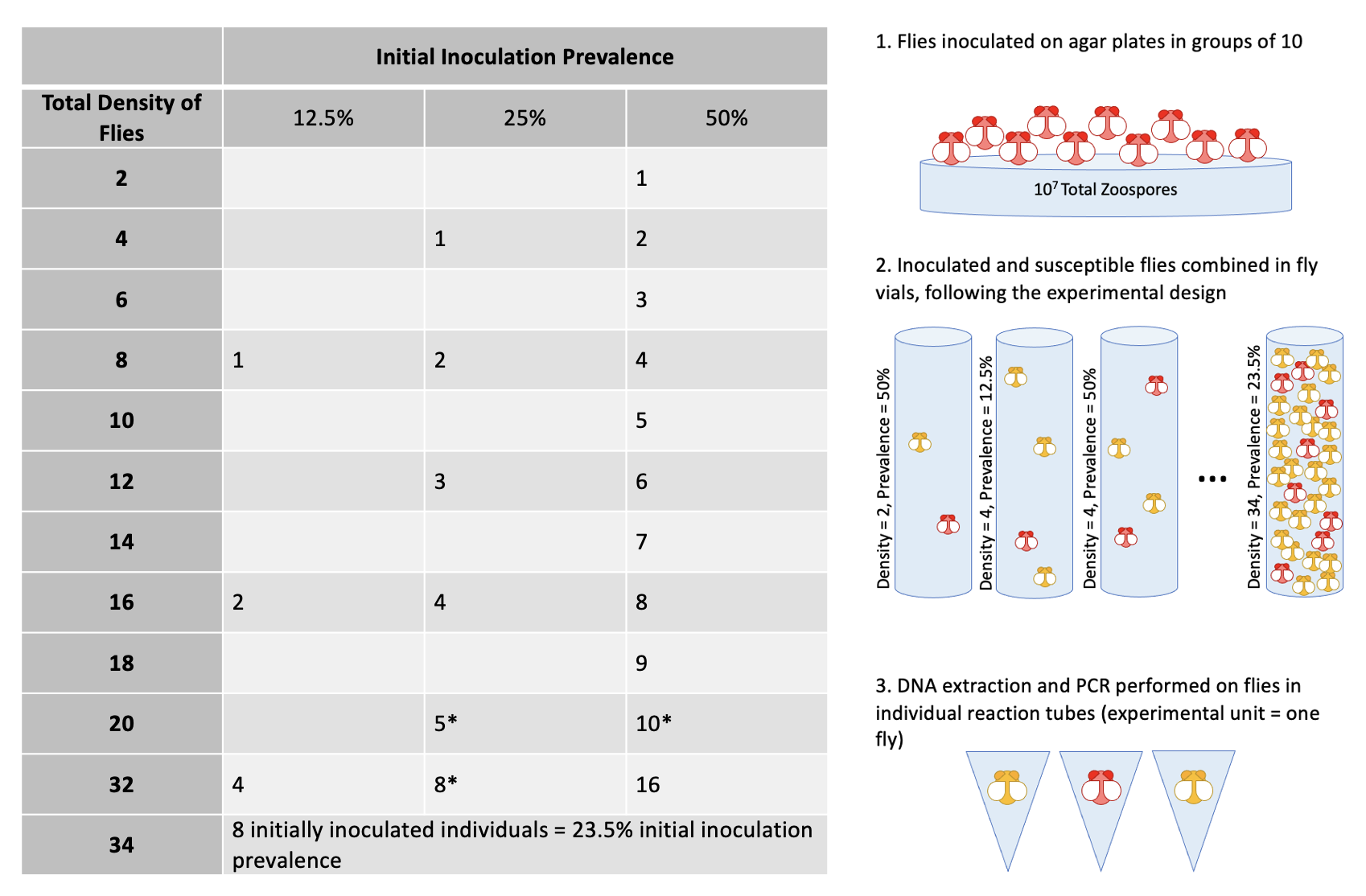

Supplement: S2 Fig — Experimental design and table were adapted from Tompros et al. 2021. The table on the left shows the total density of flies and the total number of inoculated flies in each vial. Asterisks denote treatments that were lost. In the experimental flow on the right, inoculated flies are represented in red and susceptible flies in yellow. Color indicates inoculation status only, and flies were not marked during the experiment. (PNG) [file pone.0307833.s003.png]
