# Supplementary material for: Drosophila melanogaster as a model arthropod carrier for the amphibian chytrid fungus Batrachochytrium dendrobatidis
Source: PLoS One. 2024 Jul 24;19(7):e0307833. doi: 10.1371/journal.pone.0307833 (PMC11268706; doi:10.1371/journal.pone.0307833)
Supplement: S1 Table — (DOCX) [file pone.0307833.s004.docx]

**Supporting Table 1.** Fruit fly media recipe treatments

| Recipe | Vinegar | Active Dry Yeast | Instant Potato Flakes | Powdered Sugar | Brewer’s Yeast | Warm, Sterile Deionized Water |
| --- | --- | --- | --- | --- | --- | --- |
| LV | None | 0.5 g | 40 g | 5 g | 5 g | 50 mL |
| LY | 0.5 mL | None | 40 g | 5 g | 5 g | 50 mL |
| MV | 1 mL | 0.5 g | 40 g | 5 g | 5 g | 50 mL |
| MY | 0.5 mL | 1 g | 40 g | 5 g | 5 g | 50 mL |
| New | 0.5 mL | 0.5 g | 40 g | 5 g | 5 g | 50 mL |
| Joshs (Josh’s Frog’s Fruit Fly Media) | Prepared following package directions | | | | | |
