# Supplementary material for: Drosophila melanogaster as a model arthropod carrier for the amphibian chytrid fungus Batrachochytrium dendrobatidis
Source: PLoS One. 2024 Jul 24;19(7):e0307833. doi: 10.1371/journal.pone.0307833 (PMC11268706; doi:10.1371/journal.pone.0307833)
Supplement: S2 Table — Table rows and columns give treatments, while cell values indicate the number of experimental units (one vial or five flies, their DNA pooled) in that treatment. (DOCX) [file pone.0307833.s005.docx]

**Supporting Table 2:** Inoculation experiment. Table rows and columns give treatments, while cell values indicate the number of experimental units (one vial or five flies, their DNA pooled) in that treatment.

| **Day** | **Male Control (water)** | **Male Treatment (10^6^ zsps/fly)** | **Male Heat-Killed (10^6^ zsps/fly)** | **Female Control (water)** | **Female Treatment (10^6^ zsps/fly)** | **Female Heat-Killed (10^6^ zsps/fly)** |
| --- | --- | --- | --- | --- | --- | --- |
| **1** | 3 | 3 | 2 | 3 | 3 | 2 |
| **2** | 3 | 3 | 2 | 3 | 3 | 2 |
| **3** | 3 | 3 | 2 | 3 | 3 | 2 |
| **4** | 3 | 3 | 2 | 3 | 3 | 2 |
| **5** | 3 | 3 | 2 | 3 | 3 | 2 |
