# Supplementary material for: Drosophila melanogaster as a model arthropod carrier for the amphibian chytrid fungus Batrachochytrium dendrobatidis
Source: PLoS One. 2024 Jul 24;19(7):e0307833. doi: 10.1371/journal.pone.0307833 (PMC11268706; doi:10.1371/journal.pone.0307833)
Supplement: S3 Table — Table rows and columns give treatments, while cell values indicate the number of experimental units (one vial or five flies, their DNA pooled) used in that treatment. (DOCX) [file pone.0307833.s006.docx]

**Supporting Table 3:** Dosage dependency experiment. Table rows and columns give treatments, while cell values indicate the number of experimental units (one vial or five flies, their DNA pooled) used in that treatment.

| **Bd Concentration** | **Male Washed** | **Male Unwashed** | **Female Washed** | **Female Unwashed** |
| --- | --- | --- | --- | --- |
| **0 zsps/fly** | 2 | 2 | 2 | 2 |
| **10^3^ zsps/fly** | 2 | 2 | 2 | 2 |
| **10^4^ zsps/fly** | 2 | 2 | 2 | 2 |
| **10^5^ zsps/fly** | 2 | 2 | 2 | 2 |
| **10^6^ zsps/fly** | 2 | 2 | 2 | 2 |
