# Supplementary material for: Drosophila melanogaster as a model arthropod carrier for the amphibian chytrid fungus Batrachochytrium dendrobatidis
Source: PLoS One. 2024 Jul 24;19(7):e0307833. doi: 10.1371/journal.pone.0307833 (PMC11268706; doi:10.1371/journal.pone.0307833)
Supplement: S4 Table — Table rows and columns give treatments, while cell values indicate the number of flies used in that treatment. Flies were inoculated in groups of five, and held in fly vials for observation in groups of five. (DOCX) [file pone.0307833.s007.docx]

**Supporting Table 4:** Mortality assay experiment. Table rows and columns give treatments, while cell values indicate the number of flies used in that treatment. Flies were inoculated in groups of five, and held in fly vials for observation in groups of five.

|  | **Bd Positive (10^6^ zsps/fly)** | **Bd Supernatant** | **Bd Negative (water)** |
| --- | --- | --- | --- |
| **Male** | 20 | 20 | 20 |
| **Female** | 20 | 20 | 20 |
